# Ambulant Small-Pox

**Published:** 1900-06-02

**Authors:** 


### AMBULANT SMALL-POX.

Two men travelled from Russia, where small-pox is prevalent, going *viâ* Queenborough, St. Pancras, and Manchester to Stalybridge, in Lancashire, where one of them was found to be suffering from small-pox of so malignant a type that he died there the next morning. The other man was afterwards attacked, and was removed to the Bolton Infectious Diseases Hospital. The important point, however, is that it has been ascertained that all of the four people who travelled with these two from St. Pancras to Manchester have already been attacked with small-pox. What has occurred to those who were on board the boat with them, or in the carriage with them between Queenborough and London, does not seem to have been as yet discovered, but it is clear that they ran considerable risk, and it will be very interesting to see what sort of a trail of infection a man travelling in this way while in the fully infectious stage of such a disease can leave behind him.

